# IMVAMUNE^®^ and ACAM2000^®^ Provide Different Protection against Disease When Administered Postexposure in an Intranasal Monkeypox Challenge Prairie Dog Model

**DOI:** 10.3390/vaccines8030396

**Published:** 2020-07-20

**Authors:** M. Shannon Keckler, Johanna S Salzer, Nishi Patel, Michael B Townsend, Yoshinori J Nakazawa, Jeffrey B Doty, Nadia F Gallardo-Romero, Panayampalli S Satheshkumar, Darin S Carroll, Kevin L Karem, Inger K Damon

**Affiliations:** 1 Poxvirus and Rabies Branch, Division of High-Consequence Pathogens and Pathology, Centers for Disease Control and Prevention, U.S. Department of Health and Human Services, Atlanta, GA 30333, USA; hbq0@cdc.gov (M.S.K.); hio7@cdc.gov (J.S.S.); fhw6@cdc.gov (N.P.); gbu3@cdc.gov (M.B.T.); inp7@cdc.gov (Y.J.N.); uwb7@cdc.gov (J.B.D.); hfa5@cdc.gov (N.F.G.-R.); xdv3@cdc.gov (P.S.S.); zuz4@cdc.gov (D.S.C.); kdk6@cdc.gov (K.L.K.); 2 Clinical and Environmental Microbiology Branch, Division of Healthcare Quality Promotion, Centers for Disease Control and Prevention, U.S. Department of Health and Human Services, Atlanta, GA 30333, USA; 3 Bacterial Special Pathogens Branch, Division of High-Consequence Pathogens and Pathology, Centers for Disease Control and Prevention, U.S. Department of Health and Human Services, Atlanta, GA 30333, USA; 4 Respiratory Diseases Branch, Division of Bacterial Diseases, Centers for Disease Control and Prevention, U.S. Department of Health and Human Services, Atlanta, GA 30333, USA; 5 Office of the Director, National Center for Emerging and Zoonotic Diseases, Centers for Disease Control and Prevention, U.S. Department of Health and Human Services, Atlanta, GA 30333, USA; 6 Office of the Director, Center for Global Health, Centers for Disease Control and Prevention, U.S. Department of Health and Human Services, Atlanta, GA 30333, USA

**Keywords:** monkeypox virus, postexposure vaccination, smallpox vaccines, prairie dog model

## Abstract

The protection provided by smallpox vaccines when used after exposure to Orthopoxviruses is poorly understood. Postexposu re administration of 1st generation smallpox vaccines was effective during eradication. However, historical epidemiological reports and animal studies on postexposure vaccination are difficult to extrapolate to today’s populations, and 2nd and 3rd generation vaccines, developed after eradication, have not been widely tested in postexposure vaccination scenarios. In addition to concerns about preparedness for a potential malevolent reintroduction of variola virus, humans are becoming increasingly exposed to naturally occurring zoonotic orthopoxviruses and, following these exposures, disease severity is worse in individuals who never received smallpox vaccination. This study investigated whether postexposure vaccination of prairie dogs with 2nd and 3rd generation smallpox vaccines was protective against monkeypox disease in four exposure scenarios. We infected animals with monkeypox virus at doses of 10^4^ pfu (2× LD_50_) or 10^6^ pfu (170× LD_50_) and vaccinated the animals with IMVAMUNE^®^ or ACAM2000^®^ either 1 or 3 days after challenge. Our results indicated that postexposure vaccination protected the animals to some degree from the 2× LD_50_, but not the 170× LD_5_ challenge. In the 2× LD_50_ challenge, we also observed that administration of vaccine at 1 day was more effective than administration at 3 days postexposure for IMVAMUNE^®^, but ACAM2000^®^ was similarly effective at either postexposure vaccination time-point. The effects of postexposure vaccination and correlations with survival of total and neutralizing antibody responses, protein targets, take formation, weight loss, rash burden, and viral DNA are also presented.

## 1. Introduction

Although natural infection with variola virus (VARV), the causative agent of smallpox, was eliminated in 1980 as a result of mass vaccination campaigns, followed by surveillance and vaccine containment approaches, [1] the possible regeneration of extinct viruses through synthetic biology, as with horsepox virus (HPXV), renews concerns regarding bioterrorism potential. In addition, orthopoxviruses (OPXV) such as monkeypox (MPXV), vaccinia (VACV), and cowpox (CPXV) remain global emerging zoonotic infectious diseases [2,3,4,5,6,7]. Historically, smallpox—and presumably other OPXV outbreaks—were mitigated by high levels of smallpox vaccination coverage stemming from the eradication program [8]. However, routine smallpox vaccination was discontinued in the U.S. in 1972 and ceased globally in 1984 [9,10], leaving over 50% of the world’s population without vaccine-induced immunity to OPXV-related disease and the remainder with waning immunity of varying degrees. In addition, smallpox vaccination with 2nd generation vaccines is contraindicated for an estimated 15–37% of the U.S. population [11] due to the severe adverse events (SAEs) associated with replication-capable vaccines [12,13]. The currently licensed smallpox vaccine, ACAM2000^®^, is approved for use in the U.S. for military personnel and laboratorians working with OPXV, but would not be administered to the public unless there was an ongoing OPXV outbreak [14]. The 3rd generation vaccines (i.e., IMVAMUNE^®^) are replication-impaired [15] and safer to use in vulnerable populations, and a biologics license application (BLA) for liquid-frozen formulation of IMVAMUNE^®^ (also known as MVA-BN^®^, IMVANEX^®^, and JYNNEOS^®^) has been approved by the U.S. Food and Drug Administration for use in the United States for the prevention of smallpox and monkeypox [16]. Lastly, front-line healthcare workers and emergency responders have expressed concerns about receiving smallpox vaccinations [17], indicating that noncompliance with vaccination recommendations might potentially be an issue during an OPXV-related emergency response. These factors combine to make the global population vulnerable to outbreaks of zoonotic OPXV and to the intentional release of VARV [18,19,20], demonstrating a need for additional research on the efficacy of postexposure vaccination in these scenarios.

Reviews of epidemiological data and expert opinion from the eradication period [21,22] indicate that the window for efficacy of human postexposure vaccination with 1st generation vaccines to prevent mortality and modify or eliminate morbidity of VARV infection appears to be 1–3 days after exposure. Unfortunately, 2nd and 3rd generation vaccines were not available when smallpox was endemic, so their efficacy as pre- or postexposure medical countermeasures can only be evaluated in the laboratory. Because there is no animal model for VARV, smallpox vaccines can only be tested directly against VARV using neutralization assays [23]. This makes surrogate animal models critical to smallpox vaccine research, and models using a variety of species and OPXV challenges have been developed [24,25,26,27,28,29,30,31,32,33,34,35,36,37]. However, most of these models do not have a significant incubation period between OPXV challenge and the appearance of symptoms, making postexposure therapies difficult to assess. The black-tailed prairie dog (*Cynomys ludovicianus*) manifests a MPXV infection that includes a respiratory route of infection (intranasal), a disease incubation period (6–9 days), and localized primary lesions and disseminated secondary lesions [32,38,39]. This is similar to human VARV infections and makes the prairie dog model useful for testing vaccines and therapeutics in both pre- and postexposure scenarios [40,41].

Pre-exposure vaccination with 1st, 2nd, and 3rd generation smallpox vaccines has been shown to be protective in multiple animal models using a variety of OPXV challenges [40,42,43,44,45,46,47,48]. Conversely, postexposure vaccination studies in nonhuman primate (NHP) [49] and murine animal models [50,51,52,53,54,55] have shown significantly different levels of postexposure vaccine effectiveness. While animal models of systemic OPXV infection continue to improve in their resemblance to human disease [56,57,58,59], most recent postexposure research has focused on the use of antiviral administration with or without postexposure vaccination [60,61,62,63,64,65]. This variability in previous postexposure animal study results coupled with the confounding factors in dual-treatment studies create a data gap regarding the protective effect of postexposure vaccination, which this study addressed.

This report encompasses two studies designed to determine whether postexposure vaccination with smallpox vaccines is protective when administered 1 or 3 days postexposure to prairie dogs challenged with 2× LD_50_ or 170× LD_50_ Congo Basin clade MPXV, representing low and high dose, respectively. We also present our comparisons of vaccinated and nonvaccinated group morbidity (weight loss, viral DNA, inappetence, rash burden) and immunological responses (Jennerian pustules (takes)), total and neutralizing antibody titers, protein microarrays). Lastly, we analyzed morbidity and immune responses and their correlation with survival.

## 2. Materials and Methods

### 2.1. Black-Tailed Prairie Dogs (Cynomys ludovicianus)

Eighty-six (86) wild-caught, live-trapped animals (Texas) were purchased to obtain experimental sample animals, including an equal number of males and females from a colony of similarly aged and weighted prairie dogs. This selection was performed separately by animal-care staff (50 animals for the 170× LD_50_ study and 36 for the 2× LD_50_ study). These studies were conducted approximately 2 years apart with animals from the same geographical region, with similar weights and the same female to male ratio. Animals in the 2× LD_50_ study were 2 years older than the animals used in the 170× LD_50_ study, but other animal studies in our lab have not indicated that age is a factor in the pathogenesis of MPXV [32,40,58]. Animal husbandry was performed as previously described [40,66] and in accordance with CDC Institutional Animal Care and Use Committee (IACUC)-approved protocol 1718DAMPRAC. Due to the use of MPXV as the challenge virus, the studies were executed in an Animal Biosafety Level 3 Enhanced (ABSL3E) laboratory where trained personnel performed all work. The principal investigators and animal caretakers observed each animal daily to ensure that strict euthanasia criteria were applied throughout the study. Any animal that became unresponsive to touch, lost 25% or more of its starting body weight, or accrued a total score of 10 on the following scale was humanely euthanized: decreased activity (2 points); lethargy, unsteady gait, and inappetence (3 points each); labored breathing and recumbency (5 points each). At the conclusion of each study, surviving animals were humanely euthanized. Animals were cared for and managed in accordance with IACUC policies and procedures with veterinary supervision under IACUC-approved animal protocol 2308KECPRAC-A3.

### 2.2. Viruses

The challenge virus used (Congo Basin MPXV strain, MPXV-2003-358) was collected from a 2003 outbreak of MPXV in the Republic of Congo (Centers for Disease Control and Prevention, CDC) [67]. The virus is fully sequenced and underwent two passages in African green monkey kidney cells BSC-40 (American Type Culture Collection, ATCC) prior to seed pool production. The seed pool preparations were then propagated in BSC-40 cells as previously described [68], and sucrose-cushion purified. Verification of inoculation titer for both experiments was accomplished by the same-day titration of the diluted virus aliquoted for use in challenge studies. Specific doses of virus were 4.31 × 10^6^ pfu of MPXV (170× LD_50_) and 2.25 × 10^4^ pfu of MPXV (2× LD_50_), with LD_50_ values calculated previously [69]. Live VACV vaccines based on the New York City Board of Health (NYCBOH) vaccinia strain Dryvax^®^ (Wyeth) and its clonal derivative ACAM2000^®^ (Acambis) as well as a Modified Vaccinia Ankara (MVA) strain-based vaccine IMVAMUNE^®^ (Bavarian-Nordic) were obtained and reconstituted and stored in accordance with their specific package inserts.

### 2.3. MPXV Virus Challenges

For the 170× LD_50_ study, 50 animals were distributed into 10 age–weight–sex matched groups as follows: Dryvax^®^ Day 1 (*n* = 5), Dryvax^®^ Day 3 (*n* = 5), ACAM2000^®^ Day 1 (*n* = 5), ACAM2000^®^ Day 3 (*n* = 5), IMVAMUNE^®^ Day 1 (*n* = 5), IMVAMUNE^®^ Day 3 (*n* = 5), Unvaccinated Day 1 (*n* = 5), Unvaccinated Day 3 (*n* = 5), Uninfected Day 1 (*n* = 5), and Uninfected Day 3 (*n* = 5). All animals were anesthetized using 5% isoflurane gas anesthetic to effect in a chamber, with maintenance of anesthesia by 2% isoflurane via nosecone and with the animal on a heating block to maintain core temperature in accordance with IACUC-approved protocols. Under anesthesia, 40 animals were challenged with 4.31 × 10^6^ pfu of MPXV (170× LD_50_) in 10 µL PBS and 10 animals were mock-challenged with 10 µL PBS alone. Nasal inoculation was performed by administering 5 µL of virus in PBS or PBS only into each nare of sedated animals. One animal from the Dryvax^®^ Day 3 group did not recover from anesthesia and was removed, leaving the Dryvax^®^ Day 3 group with only four animals. For the 2× LD_50_ study, the Dryvax^®^ groups were removed due to discontinuation of the vaccine. In addition, based on our experience with uninfected animals and mock vaccination in previous studies, we elected to (1) remove the uninfected group, (2) reduce the unvaccinated control group to four animals, and (3) use this group as the control for both the 1 day and 3 day postvaccination groups. This allowed us to reduce our animal use to 36 while increasing the size of the ACAM2000^®^ and IMVAMUNE^®^ groups (*n* = 8) and therefore the power of our experiment. All groups—Unvaccinated (*n* = 4), ACAM2000^®^ Day 1 (*n* = 8), ACAM2000^®^ Day 3 (*n* = 8), IMVAMUNE^®^ Day 1 (*n* = 8), and IMVAMUNE^®^ Day 3 (*n* = 8)—were challenged with 2.25 × 10^4^ pfu of MPXV (2× LD_50_) in the same manner as was done in the 170× LD_50_ study.

### 2.4. Post-Exposure Smallpox Vaccination

All vaccinations were administered following the manufacturer’s package inserts for human dose and route of administration. In the 170× LD_50_ study, anesthetized animals in the Dryvax^®^ and ACAM2000^®^ groups were inoculated on Day 1 or Day 3 postchallenge with a single dose of 2 × 10^5^ pfu of either Dryvax^®^ vaccine or ACAM2000^®^ vaccine in 10 µL of PBS via multiple punctures (15 strikes) between the shoulder blades using a tuberculin needle. Animals in the IMVAMUNE^®^ groups were given a single dose of 1 × 10^8^ tissue-culture infectious dose (TCID) of vaccine in 500 µL administered subcutaneously between the shoulder blades on Day 1 or Day 3 postchallenge. The unvaccinated controls were inoculated with 10 µL of PBS via multiple punctures (15 strikes) between the shoulder blades as a mock vaccination on Day 1 or Day 3 postchallenge. The uninfected group was not mock-vaccinated. In the 2× LD_50_ challenge, the 32 vaccinated animals were vaccinated as described above, but unvaccinated controls were not mock-vaccinated, as we had previously seen no evidence that mock-vaccination had any effect and these four animals were serving as a control group for both vaccines (ACAM2000^®^ and IMVAMUNE^®^) at both vaccination time-points (Day 1 and Day 3).

### 2.5. Weight, Body Condition, and Inappetence Measurements

The principal investigators photographed and weighed each animal under anesthesia every 2–4 days during the study. In addition, a body-condition score (BCS) was assigned to each animal every 2–4 days using the following veterinarian-defined semi-quantitative categories: 1 = Emaciated, 2 = Thin, 3 = Ideal, 4 = Stout, 5 = Obese. The BCS was determined by physical palpitation and observation of the animal for fat layers, abdomen distention, and hip, rib, or spine protrusion. Pictures of each animal taken at the time of observation were randomly examined during subsequent data analysis to ensure accuracy and to reduce bias in reported BCSs. Inappetence was quantified by measuring daily Monkey Biscuit (Exotic Nutrition, Newport News, VA, USA) and apple slice consumption.

### 2.6. Rash Burden Measurements

The principal investigators also observed nasal involvement and counted all bodily poxvirus lesions on anesthetized animals every 2–4 days. A nasal involvement score was assigned by visually examining nasal and oral areas and applying the following semi-quantitative scale: 1 = mild inflammation localized to the nares with one or more lesions; 2 = moderate inflammation with slight spread to rest of nasal area with one or more lesions; 3 = severe inflammation with spread to rest of nasal and oral area with multiple lesions; and 4 = draining lesions with or without secondary bacterial inflammation and involvement of both nasal and oral area. Body lesion counts were obtained using a visual and tactile examination of the animal for poxvirus lesions. For animals vaccinated with 1st or 2nd generation vaccines, the “take”—or Jennerian pustule—was also measured and photographed. Pictures of each animal taken at the time of observation were randomly examined during subsequent data analysis to ensure accuracy and to reduce bias of reported counts.

### 2.7. Blood and Oral Swab Sampling

For blood collection from anesthetized animals, the hind limb/groin area was sprayed with 70% isopropanol and a 28 gauge needle was used to collect ~1 mL of blood from the saphenous or femoral vein every 2–4 days. The blood was distributed to a tube containing ethylenediaminetetraacetic acid (EDTA) (Fisher Scientific) and to a serum-separation tube (Fisher Scientific) for subsequent assays. To obtain oral swab samples, a sterile cotton swab was passed over the oral cavity 10 times (2 passes each on tongue, right, left, top and bottom of oral cavity) and placed in a microcentrifuge tube for subsequent assays.

### 2.8. OPXV-Generic Quantitative PCR (qPCR)

Blood and swab samples were assayed by qPCR targeting the conserved *OPXV E9L* (DNA polymerase) gene as previously described [70]. VACV DNA was used as the positive control and a cycle threshold (CT) standard curve was generated to extrapolate fg/mL from CT values (R^2^ = 0.9901). qPCR results in fg/mL were converted to genome equivalents/mL using the conversion factor of 1 fg = 50 genome equivalents [71]. It was previously demonstrated that qPCR detection of viral DNA was significantly more sensitive than detection of pfu [68].

### 2.9. Enzyme-Linked Immunosorbent Assays (ELISAs)

A modified version of the ELISA was used for analysis of anti-OPXV immunoglobulin types A and G [46]. Briefly, plates were coated with crude VACV Wyeth (CDC) and BSC-40 cell lysate and incubated overnight. After formalin inactivation, plates were blocked. Prairie dog sera were added and incubated. Day 0 samples were run in duplicate at four-fold dilutions from 1:50 to 1:800, and Day 7, 14, and 24 samples were run in duplicate at four-fold dilutions from 1:50 to 1:51,200. Recombinant protein A/G peroxidase conjugated (Pierce Scientific) was used for detection, and plates were developed with a peroxidase substrate and stop solution (Kirkegaard & Perry Laboratories). Absorbance was read on a spectrophotometer at 450 nm. Values reported represent the average of duplicate wells for each sample. Positive human vaccinee sera were used as assay controls. A cut-off value (COV) for each plate was determined by averaging all the values on the BSC-40 lysate half of the ELISA plate and adding 2 SD. Specimens were considered positive if the test sample’s value was above the COV. Additionally, any plate that was outside of 2 SD of the mean of all the plates for either lysate background OD, lysate background SD, or the positive control OD at 1:12150 were discarded and the samples rerun. The endpoint titer for each animal at each time-point was determined based on the highest dilution that was positive and used to calculate the geometric mean titer (GMT) and SD for each group at each time-point, and are reported on a log scale.

### 2.10. High-Content Screening–Green Fluorescent Protein (HCS-GFP) Neutralization Assay

Neutralizing antibody (NAb) titers against VACV were measured using a previously validated and described GFP-based assay [72]. Briefly, a VACV-WR strain expressing GFP was incubated with sample serum at various dilutions for an hour and then used to infect cells. Serum dilutions of 1:20–1:1280 (four-fold dilutions) for Day 0 samples and 1:50–1:51,200 (four-fold dilutions) for all other time-points were used. Following the hour incubation, the supernatant was removed and media containing 46 µg/mL arabinosylcytosine added. Plates were incubated overnight at 37 °C. After 20 h, formalin-fixed, DAPI-stained plates were sealed and run on the ArrayScan HCS Reader with target-acquisition software (Thermo Scientific). The HCS-GFP assay detects the percentage of GFP-producing responder cells (%R), and this value is then normalized to control wells to produce the relative percent responders (RPR) titer. The reported values in this manuscript are the GMT and SD of 50% RPR titers, which correlate to the inhibitory concentration that neutralizes 50% of viral infection (ID_50_) in a traditional plaque-reduction neutralization titer (PRNT) assay and are reported on a log scale. The 50% RPR titer was calculated using a modified variable slope sigmoidal equation (Hill equation, Levenberg Marquardt algorithm) using Prism 5.0 software (GraphPad Software), with “goodness of fit” to this sigmoidal curve calculated by the least-squares method and represented by R^2^. All data used in this analysis had assay-specific R^2^ values greater than 0.9000.

### 2.11. Vaccinia Virus Proteome Arrays

VACV arrays were fabricated as described previously [73]. Prairie dog sera were probed at 1:100 dilution and antibodies were visualized using Protein G - Biotin (Pierce Scientific) secondary, followed with streptavidin–Surelight P-3 conjugate (Pierce Scientific). Microarrays were scanned using Gene Pix 4100A scanner (Molecular Devices) and images were analyzed with Genepix Pro 5.0 software (Molecular Devices). Spot intensity was calculated as the median spot value minus local spot background. A secondary correction for background binding to *Escherichia coli* proteins in the reaction mixture was performed. Analysis of the reactivity of prairie dog sera to each protein on the microarray was two-fold. First, corrected intensity values were log10-transformed, and then transformed data were analyzed for fold-increase from Day 0 to 7, Day 7 to 14, and Day 0 to 14 for all 225 chip proteins. Second, we applied a 1.5-fold change cut-off value to the dataset to obtain a profile of the vaccinia virus proteome targets that reacted with animal sera for each vaccination group.

### 2.12. Statistical Analysis

Prism 5.0 (GraphPad Software) was used for statistical analyses. Our data were limited by small sample sizes and high variability which needed to be accounted for in our statistical approach. In general, to maximize the power of our analysis and to select the appropriate test to measure significance, we first analyzed each dataset for normal distribution using the Shapiro–Wilk test. If data failed the normality test (*p* < 0.05), a simple transformation (Y = sqrtY) was applied to the data to see if normality was restored. If so, analysis proceeded with parametric tests. If not, then nonparametric tests were used for further analysis. Specifically, Kaplan–Meier survival curves were analyzed using the nonparametric log-rank (Mantel–Cox) test. Weight loss data were distributed normally and were analyzed using ordinary two-way analysis of variance (ANOVA). Rash burden data were not distributed normally, so data were transformed and then analyzed with ordinary two-way analysis of variance (ANOVA). Humoral responses were normally distributed and analyzed using ordinary one- and two-way analysis of variance (ANOVA). Viral DNA in blood and oral swabs data were not distributed normally and transforming the data did not restore a normal distribution, so these data were analyzed using the nonparametric Kruskal–Wallis test. Ad hoc post-tests were used as appropriate for specific comparisons and are noted in the text. These included Sidak’s, Dunn’s, Dunnett’s, Bonferroni’s, and Tukey’s multiple comparisons tests. The proteomics data we present are descriptive and were not analyzed statistically, as the sample sizes were simply too small for the multivariate analysis that would be needed. We also determined the strength of association among assorted variables and outcomes using Fisher’s exact test instead of chi-square analysis due to small sample sizes.

## 3. Results

### 3.1. Clinical Disease Progression

**Figure 1:**
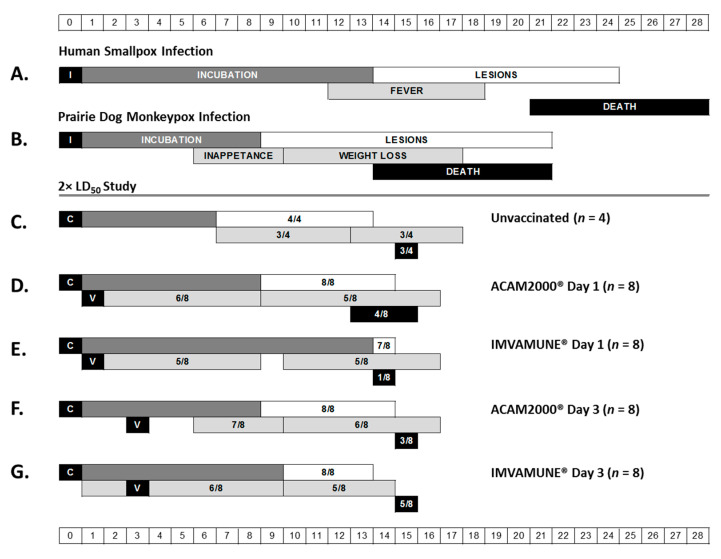
Clinical disease progression. This schematic presents a comparison of the timing of major stages of disease progression in natural human smallpox infection (**A**) and natural prairie dog monkeypox infection (**B**). The major disease stages captured include infection (I) or challenge (C), incubation (dark gray bar), febrile prodrome (light gray bars), rash (white bar), and endpoint (black bar). For human smallpox infection, the prodromal symptom represented is fever. For prairie dog monkeypox infection, the prodromal-like symptoms represented are inappetence (left light gray bar) and weight loss (right light gray bar). Endpoint represents either resolution of lesions/survival or death. The remaining rows show the results of the 2× LD_50_ study for unvaccinated animals (**C**), animals vaccinated (V) on Day 1 with ACAM2000^®^ (**D**) or IMVAMUNE^®^ (**E**), or animals vaccinated on Day 3 with ACAM2000^®^ (**F**) or IMVAMUNE^®^ (**G**). For study data, the bars (color coded as described above) represent the range of days postexposure when the onset of each stage was observed in each individual animal. The fractions in the boxes represent the number of study animals that manifested clinical signs of each stage over the total number of animals in that group.

**Figure 2:**
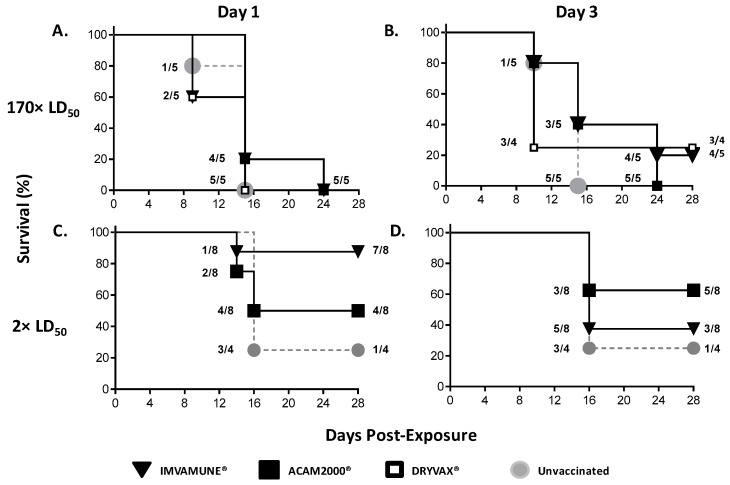
Survival benefits of post-exposure vaccination. Kaplan–Meier survival curves are shown for animals challenged with 170× LD_50_ dose and vaccinated on Day 1 (**A**) or Day 3 (**B**) postexposure with Dryvax^®^ (white squares), ACAM2000^®^ (black squares), IMVAMUNE^®^ (black triangles), or PBS (gray circles). Animals challenged with a 2× LD_50_ dose of MPXV and vaccinated 1 (**C**) or 3 (**D**) days postexposure with ACAM2000^®^ (black squares), IMVAMUNE^®^ (black triangles), or unvaccinated (gray circles) are also shown. Fractions represent the cumulative number of animals censored at each time-point over the total numbers of animals in each group. Some symbols are sized differently for clarity of superimposed data. When compared to the unvaccinated control survival curve, none of the vaccinated survival curves showed statistically significant differences by log-rank (Mantel–Cox) test (*p* > 0.05).

**Figure 3:**
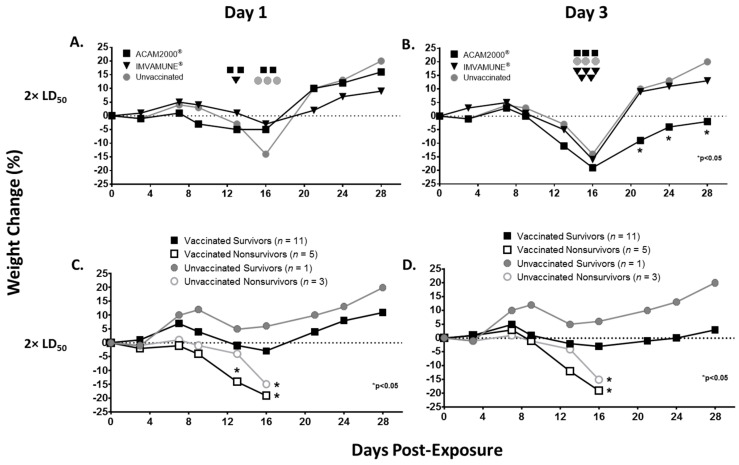
Weight loss. Group median weight changes by percentage at each time-point are shown for animals challenged with 2× LD_50_ dose of monkeypox virus and either unvaccinated (gray circles) or vaccinated 1 day postexposure (**A**) or 3 days postexposure (**B**) with ACAM2000^®^ (black squares) or IMVAMUNE^®^ (black triangles). Euthanized animals for each vaccine group are indicated on the day of euthanasia (same symbols as above). When compared to the unvaccinated animals, only Day 3 ACAM2000^®^-vaccinated animals showed statistically significant differences from Day 21 on by two-way ANOVA (*p* < 0.05). Percent weight changes are also shown for vaccinated survivors (filled black squares), vaccinated nonsurvivors (open black squares), unvaccinated survivors (filled gray circles), and unvaccinated nonsurvivors (open gray circles) vaccinated on Day 1 (**C**) or Day 3 (**D**) postexposure. When compared to the unvaccinated survivor controls, Day 1 vaccinated nonsurvivors showed significantly more weight loss on Day 13. On Day 16, both vaccinated and unvaccinated nonsurvivors showed significantly more weight loss regardless of vaccination timing by two-way ANOVA (*p* < 0.05).

**Figure 4:**
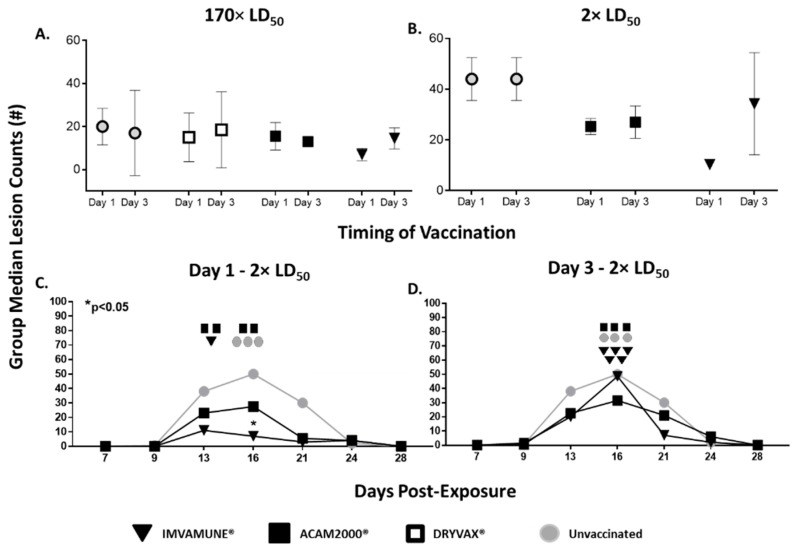
Rash burden. Group median peak (Day 16) lesion counts are shown for all animals that received 170× LD_50_ MPXV dose (**A**) and 2× LD_50_ (**B**) for both Day 1 (left symbol) and Day 3 (right symbol) for all groups—unvaccinated (gray circles), Dryvax^®^-vaccinated (white square – 170× LD_50_ study only) ACAM2000^®^-vaccinated (black squares) and IMVAMUNE^®^-vaccinated animals (black triangles). No significant differences were seen between vaccine group or vaccination timing by two-way ANOVA (*p* < 0.05). Group median secondary lesion counts at each time-point are shown for animals challenged with 2× LD_50_ dose of monkeypox virus and either unvaccinated (gray circles) or vaccinated 1 day postexposure (**C**) or 3 days postexposure (**D**) with ACAM2000^®^ (black squares) or IMVAMUNE^®^ (black triangles). Euthanized animals for each vaccine group are indicated on the day of euthanasia (same symbols as above). Only animals vaccinated with IMVAMUNE^®^ on Day 1 postexposure differed significantly from unvaccinated controls by two-way ANOVA (*p* < 0.05).

Although postexposure vaccination with any vaccine in the 170× LD_50_ challenge did not alter measured clinical scores of disease severity with respect to non-post-exposure-vaccinated animals, the time to death was delayed in a subset of PEP-vaccinated animals; additionally, the cause of death in a subset of post-exposure-vaccinated animals may have been due to secondary infections, rather than due to monkeypox infection. We mapped the disease progression of our 2× LD_50_ study animals as shown in Figure 1. The graphic shows the day of challenge, day of postchallenge vaccination, and the range of days where we initially observed the appearance of each disease stage in each group. The first two rows of this figure show the typical disease progression in smallpox-infected humans (Figure 1A) and MPXV-infected prairie dogs (Figure 1B). The remaining rows show the disease progression in all groups of 2× LD_50_ study animals for comparison (Figure 1C–G). At this dose, humane euthanasia of ill animals began between Days 13 and 16 for all groups regardless of vaccination status. The majority of animals progressed through all stages of disease progression, with prodromal symptoms in 63–100% of animals in each group and lesions in 88–100%. Mortality for unvaccinated animals (Figure 1C and Figure 2C) was 75% (3/4). Overall, vaccination was beneficial and vaccinated animals exhibited lower mortality compared to control animals (Figure 1D–G and Figure 2C,D). The lowest mortality rate of 12% (1/8) was achieved with IMVAMUNE^®^ vaccination on Day 1 postexposure (Figure 1E and Figure 2C). For other treatments, mortality rates of 62% (5/8) for animals vaccinated with IMVAMUNE^®^ on Day 3 (Figure 1G and Figure 2D), 50% (4/8) in animals vaccinated with ACAM2000^®^ on Day 1 (Figure 1D and Figure 2C), and 38% (3/8) in animals vaccinated with ACAM2000^®^ on Day 3 were observed (Figure 1F and Figure 2D). At the 2× LD_50_ dose, unvaccinated animals began exhibiting inappetence on Day 7, and animals vaccinated with ACAM2000^®^ on Day 3 began exhibiting inappetence on Day 6. All other groups showed an earlier onset of inappetence, beginning on Day 1 or Day 2. Weight loss (Figure 1 and Figure 3) for unvaccinated animals began on Day 13, while vaccinated animals began losing weight earlier (range Day 9 to Day 10), consistent with inappetence observations. Unvaccinated animals began exhibiting lesions on Day 6, while lesion development was delayed to Day 9 or 10 in most vaccinated groups. Interestingly, lesion onset in the animals vaccinated one day postexposure with IMVAMUNE^®^ was delayed until Day 14. (Figure 1 and Figure 4). 

### 3.2. Survival Benefits

Figure 2 shows Kaplan–Meier survival curves for all animal groups. For animals challenged with 170× LD_50_ MPXV and vaccinated one day postchallenge (Figure 2A), none survived with any of the post-exposure-administered vaccines (Dryvax^®^, ACAM2000^®^, and IMVAMUNE^®^). Only one animal vaccinated with Dryvax^®^ or IMVAMUNE^®^ three days after challenge survived (25% (1/4) or 20% (1/5)), respectively (Figure 2B). No postexposure vaccination had a statistically significant survival benefit at this dose (*p* > 0.05) by log-rank (Mantel–Cox) test when compared to unvaccinated animals.

For animals challenged with 2× LD_50_ MPXV, the unvaccinated animals’ survival rate was 25% (1/4). Vaccination one day postchallenge (Figure 2C) resulted in survival rates of 88% (7/8) and 50% (4/8) for IMVAMUNE^®^- and ACAM2000^®^-vaccinated animals, respectively. For animals vaccinated three days postchallenge (Figure 2D), the survival rate with ACAM2000^®^ was 62% (5/8), while the survival rate of animals vaccinated with IMVAMUNE^®^ was 38% (3/8). No postexposure vaccination had a statistically significant survival benefit at this dose (*p* > 0.05) by log-rank (Mantel–Cox) test when compared to unvaccinated animals.

### 3.3. Weight Loss

Animals challenged with 170× LD_50_ MPXV and vaccinated on Day 1 or on Day 3 postchallenge manifested no significant differences in weight loss between those receiving vaccine or not, nor between aggregated survivors and nonsurvivors irrespective of vaccination status (data not shown).

Weight loss over the course of illness in animals challenged with 2× LD_50_ MPXV is presented in Figure 3. For groups vaccinated at Day 1 (Figure 3A), weight loss for unvaccinated animals began after Day 7, reached −3% on Day 13 and dropped to −14% by Day 16. Animals vaccinated on Day 1 began weight loss at Day 7, but lost less weight, with ACAM2000^®^-vaccinated animals reaching a plateau at −5% on Day 13 and Day 16 and IMVAMUNE^®^-vaccinated animals reaching a maximum weight loss of −3% on Day 16. All groups had returned to at least original weights by Day 21. Although maximum weight loss in unvaccinated animals was 3–5 times higher than vaccinated animals on Day 16, these differences did not reach significance by two-way ANOVA.

For the animals vaccinated on Day 3 (Figure 3B), the onset of weight loss also began on Day 7 and reached its maximum on Day 16. However, unlike animals vaccinated on Day 1, those vaccinated on Day 3 showed no significant mitigation of weight loss compared to the unvaccinated animals. IMVAMUNE^®^-vaccinated animals’ weight loss was −5% at Day 13 and dropped to −16% at Day 16, while ACAM2000^®^-vaccinated animals’ weight loss was −11% at Day 13 and −19% by Day 16. The ACAM2000^®^-, but not IMVAMUNE^®^-vaccinated animals’ median weight was significantly lower than unvaccinated animals on Days 21, 24, and 28 (*p* < 0.05) by two-way ANOVA.

We analyzed weight loss data by survivors and nonsurvivors regardless of vaccination status for both Day 1 (Figure 3C) and Day 3 (Figure 3D) groups. As expected, loss of weight was associated with nonsurvival, with significant differences in median weight loss on Day 12 and 16 in nonsurviving animals compared to the single unvaccinated survivor by two-way ANOVA.

### 3.4. Rash Burden

In animals challenged with 170× LD_50_ MPXV (Figure 4A) or 2× LD_50_ MPXV (Figure 4B) there were no significant differences between peak (Day 16) group median lesion counts for ACAM2000^®^- or IMVAMUNE^®^-vaccinated animals, regardless of the day of vaccination. This was true for all sampling time-points (data not shown). In the animals challenged with 170× LD_50_ MPXV and vaccinated on Day 1 or Day 3, there were no differences between lesion counts in vaccinated and unvaccinated animals (data not shown). However, in the 2× LD50 challenge, all animals vaccinated 1 day postexposure (Figure 4C) manifested fewer secondary lesions than unvaccinated animals, with IMVAMUNE^®^-vaccinated animals having significantly fewer at Day 16 by two-way ANOVA (*p* < 0.05). For animals vaccinated on Day 3 postexposure (Figure 4D), ACAM2000^®^-vaccinated—but not IMVAMUNE^®^-vaccinated—animals’ lesion counts were lower than unvaccinated animals at all time-points, but this difference did not reach statistical significance by two-way ANOVA (*p* < 0.05).

### 3.5. Humoral Immune Response

**Figure 5:**
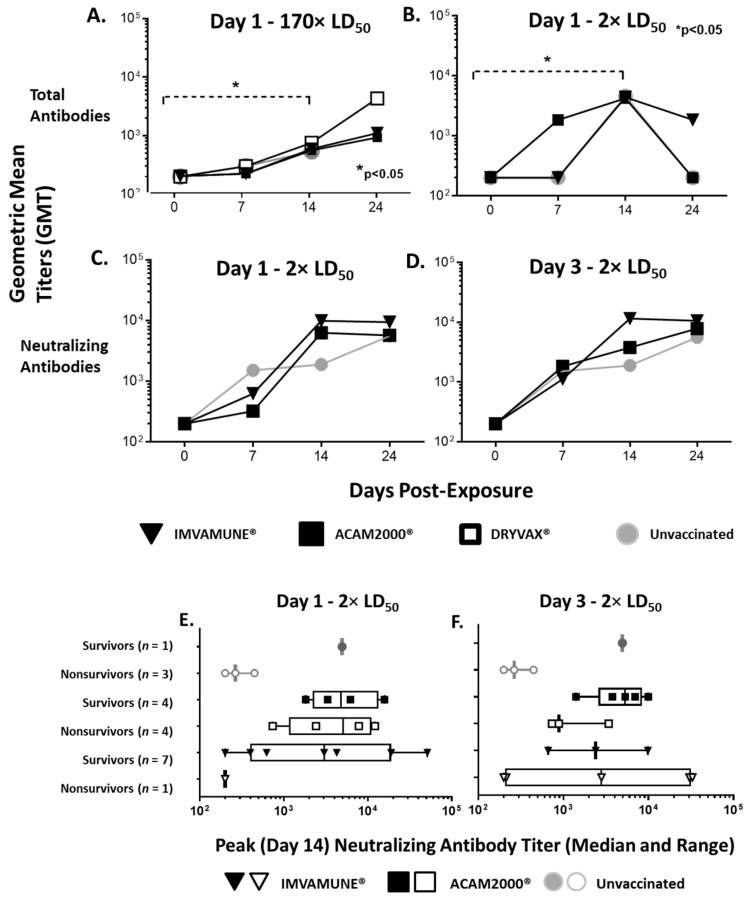
Humoral immune response. Total antibodies (geometric mean titers) at each time-point for animals challenged with 170× LD_50_ dose (**A**) or 2× LD_50_ dose (**B**) of monkeypox virus and unvaccinated (gray circles) or vaccinated 1 day postexposure with Dryvax^®^ (white squares), ACAM2000^®^ (black squares), or IMVAMUNE^®^ (black triangles) showed significant increases from Day 0 to Day 14 by two-way ANOVA (*p* < 0.05). A similar analysis of neutralizing antibody titers from animals challenged with 2× LD_50_ dose and vaccinated on Day 1 (**C**) or Day 3 (**D**) postexposure is keyed the same and showed no significant difference between unvaccinated and vaccinated animals by two-way ANOVA. Geometric mean titers from peak neutralizing antibody response (Day 14) are shown for both surviving and nonsurviving animals challenged with 2× LD_50_ dose of monkeypox virus and vaccinated 1 (**E**) or 3 (**F**) days postexposure, with no significant differences as measured by one-way ANOVA.

We compared the timing and peak geometric mean endpoint titers of total antibodies at both doses for unvaccinated animals and vaccinated animals. In animals challenged with 170× LD_50_ MPXV (Figure 5A), the temporal analysis of total antibodies showed that all groups had antibody titers below the level of detection on Day 0, with a small increase in all group titers on Day 7 and titers rising significantly from Day 0 to Day 14 by two-way ANOVA (*p* < 0.05). A similar pattern was seen in animals challenged with 2× LD_50_ MPXV (Figure 5B), with all group antibody titers being below the level of detection on Day 0 and with titers rising significantly from Day 0 to Day 14 by two-way ANOVA (*p* < 0.05). However, ACAM2000^®^-vaccinated animals generated a log increase in antibodies on Day 7 that was not seen in IMVAMUNE^®^-vaccinated or unvaccinated animals at the 2× LD_50_ dose and was not seen in any groups at the 170× LD_50_ dose (data not shown). Animals vaccinated on Day 3 showed similar total antibody kinetics (data not shown).

A similar analysis of neutralizing antibodies in the animals vaccinated 1 or 3 days postexposure in the 2× LD_50_ study is shown in Figure 5C,D. In the animals vaccinated on Day 1 (Figure 5C), the neutralizing antibody titers increased on Day 7, peaked at Day 14, and plateaued on Day 24. Unvaccinated animals showed the largest increase from Day 0 to Day 7, but plateaued from Day 7 to 14 and increased again by Day 24. For animals vaccinated on Day 3 postexposure (Figure 5D), both ACAM2000^®^- and IMVAMUNE^®^-vaccinated animals had a log higher antibody titer on Day 7 than was exhibited by the animals vaccinated one day postexposure, and the increase in neutralizing antibodies in ACAM2000^®^-vaccinated animals was less on Day 14. However, none of these differences reached statistical significance by two-way ANOVA (*p* > 0.05). For the 170× LD_50_ study, we found no significant differences in neutralizing antibodies at any time-point among any of the groups regardless of vaccination timing (data not shown).

Lastly, we organized the Day 14 peak neutralizing antibody data to compare the geometric peak mean titers of neutralizing antibody between survivors and nonsurvivors within each treatment group in the 2× LD_50_ study. For Day 1 (Figure 5E) postexposure vaccination, all surviving groups had geometric peak mean titers higher than 10^3^ on Day 14. The ACAM2000^®^-vaccinated animals that did not survive had a similar mean titer, while unvaccinated and IMVAMUNE^®^-vaccinated animals that did not survive had mean antibody levels which were a log lower. For Day 3 (Figure 5F) postexposure vaccination, the surviving group geometric mean peak titers over 10^3^ on Day 14. The IMVAMUNE^®^-vaccinated animals that did not survive had a similar mean titer, while unvaccinated and ACAM2000^®^-vaccinated animals that did not survive had peak antibody levels, which were a log lower. None of these differences reached statistical significance by one-way ANOVA.

### 3.6. Viral DNA in Blood and Oral Swabs

**Figure 6:**
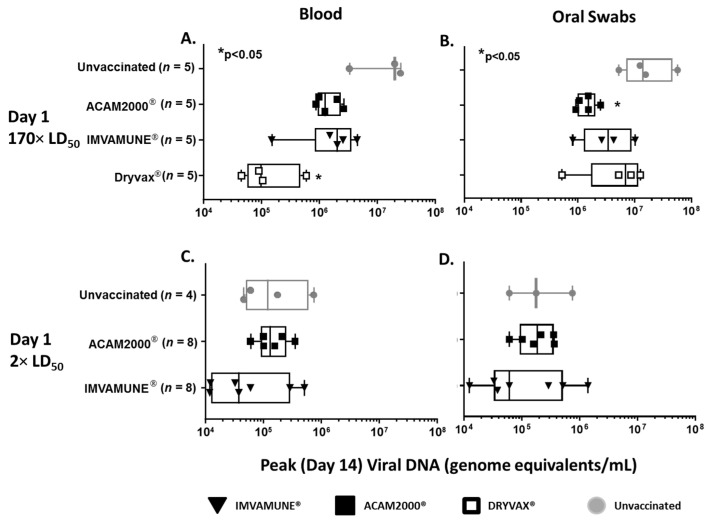
Viral DNA in Blood and Oral Swabs. Total viral DNA (genome equivalents/mL) found in blood (**A**) and oral swabs (**B**) from animals challenged with 170× LD_50_ dose of MPXV and unvaccinated (gray circles) or vaccinated 1 day postexposure with Dryvax^®^ (white squares), ACAM2000^®^ (black squares), or IMVAMUNE^®^ (black triangles). Only Dryvax^®^-vaccinated animals had significantly lower levels of viral DNA in blood and only ACAM 2000^®^-vaccinated animals had significantly lower levels of viral DNA from oral swabs by Kruskal–Wallis test (*p* < 0.05). For animals receiving a 2× LD_50_ dose, neither IMVAMUNE^®^ or ACAM2000^®^ vaccination significantly decreased viral load in blood (**C**) or oral swabs (**D**) by Kruskal–Wallis test (*p* < 0.05).

Analysis of RT-PCR results from oral swabs and blood showed that viral DNA peaked at Day 14. Animals vaccinated on Day 3 in both studies showed no differences in median peak viral DNA (data not shown). In animals vaccinated on Day 1 and challenged with 170× LD_50_ MPXV, the median peak viral DNA in blood (Figure 6A) ranged from 10^5^ to 10^7^ and was lower in all vaccinated animals, but only Dryvax^®^ vaccination resulted in significantly lower median peak viral DNA in blood when compared to unvaccinated animals. In oral swabs (Figure 6B) the range of viral DNA was 10^6^ to 10^7^ and all vaccinated animals had decreased viral DNA on Day 14. However, only ACAM2000^®^ vaccination resulted in significantly lower median peak viral DNA in oral swabs. In animals challenged with 2× LD_50_ MPXV, viral DNA in blood ranged from 10^4^ to 10^6^ (Figure 6C) and viral DNA from oral swabs (Figure 6D) ranged from 10^4^ to 10^7^, with only IMVAMUNE^®^-vaccinated animals having a lower median peak viral DNA in both blood and oral swabs when compared to unvaccinated animals, although these differences were not statistically significant by Kruskal–Wallis test.

### 3.7. Vaccinia Virus Proteome Arrays

**Figure 7:**
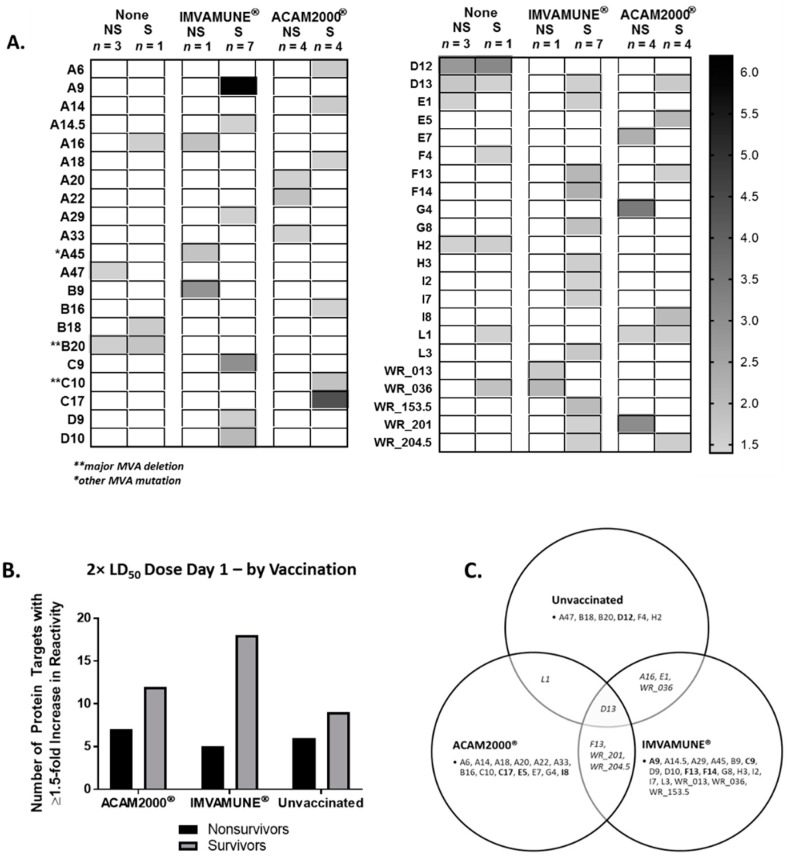
Protein targets for antibody response determined by proteome microarray. Sera from animals challenged with a 2× LD_50_ dose of monkeypox virus and vaccinated 1 day postexposure were analyzed by microarray. Proteins where fold-increases in reactivity were ≥1.5 are displayed as a heat map (**A**) with average fold-increase for unvaccinated (Columns 1 and 2), IMVAMUNE^®^-vaccinated (Columns 3 and 4), and ACAM2000^®^-vaccinated (Columns 5 and 6) nonsurvivors (odd numbered columns) and survivors (even numbered columns). The total number of protein targets with a ≥1.5 fold-increase in reactivity with animal serum is shown for all groups of animals (**B**), subgrouped by survivors (gray column) and nonsurvivors (black column). The Venn diagram (**C**) describes the overlap of protein targets with a ≥1.5 fold-increase in reactivity among the groups, with the most reactive targets per group highlighted (bold).

To better understand the differences in survival we observed in animals vaccinated on Day 1 and challenged with 2× LD_50_ MPXV, microarray proteins where fold-increases in reactivity against sera from animals challenged with 2× LD_50_ MPXV were ≥1.5 are displayed as a heat map (Figure 7A). The D12 protein had the highest fold-increase in reactivity in both surviving (3.1-fold) and nonsurviving (2.8-fold) unvaccinated animals. In IMVAMUNE^®^-vaccinated animals, the highest fold-increases were seen in A9 (6.1-fold), C9 (3.0-fold), F14 (2.2-fold), and F13 (2.1-fold) for surviving animals, and B9 (2.9-fold) and WR_036 (2.1-fold) in the single nonsurviving animal. In ACAM2000^®^-vaccinated animals that survived, the highest fold-increases in protein reactivity were seen in C17 (4.4-fold), E5 (2.1-fold), and I8 (2.0-fold) compared to the highest fold-increases in nonsurviving animals found in G4 (3.5 fold), WR_201 (3.1-fold), and E7 (2.3-fold). The number of separate protein targets identified for nonsurvivors vs. survivors of each vaccination group is shown in Figure 7B. In general, surviving animal sera were reactive with more protein targets than nonsurviving animal sera, with seven targets recognized by ACAM2000^®^ nonsurvivor sera and 12 targets for survivor sera. For IMVAMUNE^®^-vaccinated animals, the nonsurvivor vs. survivor targets were 5 vs. 18, and for unvaccinated animals were 6 vs. 9 targets. Figure 7C shows the targets recognized by unvaccinated animals that overlapped with targets recognized from IMVAMUNE^®^-vaccinated animals (A16, E1, and WR_036), ACAM2000^®^-vaccinated animals (L1), or both (D13). Animals vaccinated with IMVAMUNE^®^ or ACAM2000^®^ both had antibodies which reacted to F13, WR_201, and WR_204.5, while all other targets were specific to each animal group.

### 3.8. Jennerian Pustules (“Takes”)

**Figure 8:**
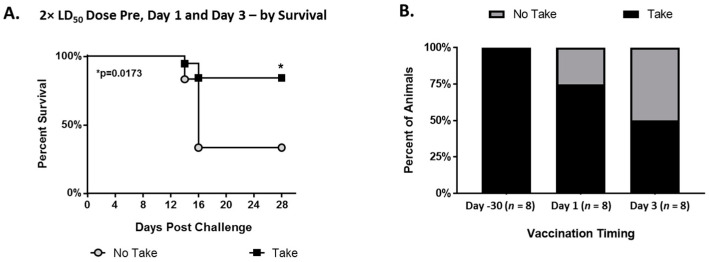
Jennerian pustule “takes”. Kaplan–Meier survival curves for animals from previous and current studies challenged with 2× LD_50_ dose of monkeypox virus and vaccinated with ACAM2000^®^ or Dryvax^®^ either 30 days prior to challenge or 1 or 3 days postexposure (**A**) and which manifested a take at the vaccine site (black squares) or did not (gray circles). Survival was significantly higher for animals who manifested a take by log-rank (Mantel–Cox) test (* *p* > 0.05). Comparison of multiple studies with animals challenged with 2× LD_50_ dose of monkeypox virus and vaccinated with ACAM2000^®^ or Dryvax^®^ at Day −30 pre-exposure, 1 day postexposure, or 3 days postexposure (**B**), showing percentage of animals that manifested a take (brown) or did not manifest a take (black).

Using historical data from a previous study (37and the data from this study, we analyzed the appearance of “takes” in various vaccination-timing scenarios. Animals which exhibited a “take” were significantly more likely to survive than animals that did not have a demonstrable “take” (*p* = 0.0173) (Figure 8A). In pre-exposure vaccination, 100% of animals formed a “take”, which dropped to ~75% with Day 1 postexposure vaccination and then to ~50% with Day 3 postexposure vaccination (Figure 8B).

### 3.9. Relative Risk Analysis

**Figure 9:**
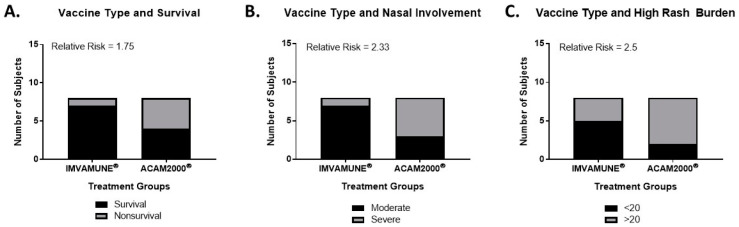
Relative risk analysis. In animals challenged with 2× LD_50_ MPXV dose, the relative risk of survival (**A**) and mitigation of primary (**B**) and secondary lesions (**C**) are shown for IMVAMUNE^®^ (left) and ACAM2000^®^ (right) vaccination. No differences between the vaccines reached significance by Fisher’s exact test.

Relative risk was calculated to compare outcomes based on specific vaccine, with results presented in Figure 9. In 2× LD_50_ MPXV challenge studies, vaccination on Day 1 with IMVAMUNE^®^ was associated with a 75% increase in the probability of survival (Figure 9A) when compared to ACAM2000^®^. IMVAMUNE^®^ vaccination on Day 1 was also associated with a 133% and 150% increase in the probability of mitigated primary and secondary lesions, respectively (as measured by nasal involvement and pox lesion burden (Figure 9B,C). However, none of these differences were statistically significant by Fisher’s exact test.

## 4. Discussion

This study evaluated the efficacy and potential correlates of protection from postexposure smallpox vaccination in a small animal model of systemic OPXV infection. In this case, postexposure refers to postinfectious challenge with virus, whereas in epidemiological studies, postexposure may not necessarily be the same as postinfection. The great similarity of the disease incubation period in the prairie dog model to human systemic orthopoxvirus infections (Figure 1) helps to overcome some of the more extreme extrapolation problems found in postexposure vaccination studies using other animal models that lack an extended incubation period. The use of the high challenge dose in our first study was based on a previous study showing full protection of Dryvax^®^, ACAM2000^®^, and IMVAMUNE^®^ pre-exposure vaccination against a 170× LD_50_ MPXV challenge in this animal model [40]. Post-exposure vaccination in this model was not protective against death at this challenge dose (Figure 2), which was similar to findings from a nonhuman primate animal study where administration of 1st generation vaccines 24 h after a 1 × 10^7^ pfu MPXV (100× LD_50_) challenge was not protective [49]. Interestingly, in both 170× LD_50_ challenges, all unvaccinated animals succumbed to MPXV disease by Day 15. However, of the animals vaccinated one day postexposure, 20% (1/5) of animals in both the IMVAMUNE^®^ and ACAM2000^®^ vaccine groups survived until Day 24. In animal groups vaccinated three days postexposure, 40% (2/5) of animals from both the ACAM2000^®^- and IMVAMUNE^®^-vaccinated animal groups survived until Day 24, with one Dryvax^®^- and one IMVAMUNE^®^-vaccinated animal recovering fully. Clinical observations and necropsies indicated that the animals that survived until Day 24 appeared to succumb to secondary respiratory bacterial infections (based on veterinarian assessment of clinical signs), indicating that these animals might have recovered from the MPXV challenge had the bacterial infection been treated. These data are useful for emergency planning, as viral doses might be higher in a bioterrorism event.

Between the 170× LD_50_ study and the 2× LD_50_ study, Dryvax^®^ was discontinued for use in humans so was removed from the second study. Using a lower 2× LD_50_ MPXV challenge was based on literature showing postexposure vaccination protection in mice at a 3× LD_50_ ECTV challenge [54], and this dose is likely more relevant to the use of smallpox vaccines in a natural outbreak. Similar to the ectromelia studies, we saw some protection against mortality if either vaccine was administered either 1 or 3 days postvaccination (Figure 2). Interestingly, the ACAM2000^®^ vaccine offered very similar benefit when administered on either Day 1 or Day 3, but IMVAMUNE^®^ protection was much greater when administered on Day 1 than on Day 3, despite IMVAMUNE^®^-induced antibody levels being as high as or higher than antibodies found in ACAM2000^®^-vaccinated animals (Figure 5). This is consistent with neutralizing antibodies not being sufficient for total protection from OPXV infections and may be related to the presence of a cross-reactive IgM response. It would be expected that the IgM response would be greater at Day 3 postchallenge than Day 1, and might neutralize the replication-deficient IMVAMUNE^®^ vaccine before it can effectively establish a strong cell-mediated immune response [73,74]. This is supported by the demonstrated dependence of MVA on cell-mediated immunity to protect mice and macaques from death when given immediately before or after challenge [48,50,51,52,63]. These findings have relevance in understanding the effectiveness of postexposure vaccination in populations that are contraindicated for ACAM2000^®^ [36,68]. Speculatively, this effect could also be associated with a stronger innate immune response post-IMVAMUNE^®^-vaccination, and we did not measure innate immune response markers in this study.

Approximately 10,000,000 Americans are immunocompromised, constituting one of multiple subpopulations that are contraindicated for ACAM2000^®^ vaccination [36,52]. The vulnerability of these populations makes IMVAMUNE^®^’s ability to modify disease of special importance. In our study, we saw evidence for disease modification by IMVAMUNE^®^ in protection against weight loss (Figure 3) and rash burden (Figure 4) when administered on Day 1 (but not Day 3) postexposure, and this protection was similar to, or better than what was seen with ACAM2000^®^ vaccination. Results of viral DNA in blood and oral swabs (Figure 6) showed that IMVAMUNE^®^-vaccinated animals had lower viral DNA in blood and oral swabs when compared to unvaccinated animals. ACAM2000^®^ vaccination resulted in lower viral DNA in blood and oral swabs than IMVAMUNE^®^ at the 170× LD_50_ dose, but not at the 2× LD_50_ dose, which is of relevance to bioterrorism medical countermeasure planning. Relative risk analysis of survival and primary and secondary lesions by vaccines indicates that, at a 2× LD_50_ dose, IMVAMUNE^®^ vaccination on Day 1 increases the probability of survival and leads to severe primary and secondary lesions more than vaccination with ACAM2000^®^, although both vaccines increased survival and lessened rash burden when compared to unvaccinated animals.

Our data also showed that antibodies in sera from unvaccinated animals were generally less reactive with an array of VACV proteins than sera from animals vaccinated 1 day postchallenge (Figure 7), indicating that breadth of antibody response may play a role in survival. Interestingly, although the overall reactivity profile was similar to previous microarray data, with previously reported OPXV antibody targets showing the highest reactivity (A26, A11, I1, A17, H3, D13, A34, WR-169, C23, D8, A56, A36, A6, A27, A33, A21, O1, and A29) [73], our analysis using group-averaged fold-increases identified that the number and identity of protein targets differed between ACAM2000^®^- and IMVAMUNE^®^-vaccinated animals, which was different from pre-exposure studies showing similar reactivity profiles between Dryvax^®^ and MVA [74,75]. Of the protein targets identified in this study, the most reactive protein targets were specific for a single group of animals. These immunodominant targets included D12 for unvaccinated animals; C17, E5, and I8 for ACAM2000^®^-vaccinated animals; and A9, C9, F13, and F14 for IMVAMUNE^®^-vaccinated animals. These observations differed from array data seen in MVA or VACV pre-exposure vaccination of humans, rabbits, and macaques [74,75]. However, D12 and C9 have been shown to be immunoreactive in vaccinated humans by Elispot analysis [76]. Results from this study indicate that the humoral response against OPXV exposure may modified by postexposure vaccination and needs further characterization.

The historical use of a “take” as a surrogate for protection is consistent with our data showing that surviving animals had a significantly higher incident of “takes” than did nonsurviving animals (Figure 8) in various vaccination scenarios. In addition, the decrease in “takes” seen in postexposure vaccination compared to pre-exposure vaccination was consistent with the hypothesis that postexposure vaccination can be affected by the initial IgM response to MPXV exposure. While we do not know the importance or mechanism of these differences, these data could contribute to future investigations of the feasibility of vaccines specific for postexposure use in targeted vaccination [52,55,56].

## 5. Conclusions

In summary, these studies contribute novel data to the body of knowledge regarding postinfection vaccination against systemic OPXV disease, and support the use of 2nd and 3rd generation vaccines like ACAM2000^®^ and IMVAMUNE^®^, respectively, as a postexposure medical countermeasure against an intentional OPXV release or zoonotic outbreak. In a smallpox event, postexposure vaccination would include the vaccination of contacts and their respective close household contacts, which would include both infected and uninfected persons. This study indicated that the use of postexposure vaccination, can provide benefit against disease severity and in some instances may prevent death in infected persons These results are consistent with the generally accepted 3 day window of postexposure vaccination effectiveness for replication competent vaccines and provides data about the efficacy of nonreplicating vaccines in decreasing mortality and morbidity after orthopoxvirus infection. One limitation to this study is the potentially confounding variable of repeated anesthesia. The animals were anesthetized every 2–4 days during the study and the effect of that on vaccination and disease progression is unclear. This study also demonstrates the need for more robust studies of innate, humoral, and cell-mediated immune responses to replicating and nonreplicating vaccines in order to better understand how smallpox vaccines protect against disease. This information could be useful for developing medical countermeasure policies on the use of postexposure vaccination.
